# Generating Political Commitment for Regulatory Interventions Targeting Dietary Harms and Poor Nutrition: A Case Study on Sugar-Sweetened Beverage Taxation in Australia

**DOI:** 10.34172/ijhpm.2021.174

**Published:** 2021-12-22

**Authors:** Tristan Dry, Phillip Baker

**Affiliations:** ^1^Public Health, School of Medicine, Griffith University, Gold Coast, QLD, Australia.; ^2^Institute for Physical Activity and Nutrition, Deakin University, VIC, Geelong, Australia.

**Keywords:** Australia, Food Policy, Nutrition Policy, Political Economy, Advocacy, Nutrition

## Abstract

**Background:** Sugar-sweetened beverage (SSB) taxes are an effective public health policy intervention for improving nutrition and public health. Although implemented in over 50 jurisdictions worldwide, this intervention remains vastly underutilised, and in Australia political commitment for such a tax is low. The aim of this study is to understand the politics of SSB taxation in Australia, what factors have constrained political commitment for a tax, and what might enable such commitment in future.

**Methods:** We adopted a case study design, guided by a theoretical framework developed from the political economy of nutrition literature. Data were collected from 16 interviews with informants from multiple sectors, supported by media articles, journal articles, and grey literature. Data were coded and organized by thematic analysis, and synthesised into the final results.

**Results:** Nutrition actors have made significant progress in generating commitment for a SSB tax by producing relevant evidence, raising awareness, advocating for action, employing resonating frames, collaborating with civil society organisations, and forming coalitions increasing their overall cohesion. Nevertheless, political commitment for a SSB tax is low and was found to be impeded by the powerful influence of the food, beverage, and sugar industries, opposition from both major Australian political parties, ideological resistance to regulation, a low quality monitoring and surveillance system for food and nutrition, and limited public advocacy. The influence of nutrition actors was also impeded by weak connections to key policy-makers and missed collaborative opportunities with pro-SSB tax organisations.

**Conclusion:** The identification of several impediments provides an explanation for why political commitment for a SSB tax is low in Australia and reveals several opportunities for how it might be generated in the future. Political commitment may come about through, for example, actions to limit the influence of industry in policy decision-making, and by strengthening the existing pro-SSB tax coalition.

## Background

**Key Messages**

** Implications for policy makers**

- A growing literature on the political economy of nutrition identifies a set of factors that enable and constrain the emergence of political commitment for the prevention of dietary harms and poor nutrition. The political commitment for nutrition framework presents a set of such factors, that were used to guide the present study.
- A major impediment to political commitment for a sugar-sweetened beverage (SSB) tax in Australia is the strong and well-connected political influence of the food and beverage industries, which employed strategic frames (eg, individuals and parents are responsible for SSB consumption), cast doubt on the public health evidence-base, promoted self-regulation, and lobbied government in an effort to undermine commitment. This was further strengthened by the importance of the sugar industry in marginal seats within the Australian electoral system, together with its vocal opposition. Ideological resistance to regulation from within government further impeded commitment.
- Strong leadership and collaboration in the nutrition community enabled commitment by generating an evidence-base, raising awareness, advocating for action, employing resonating frames (eg, negative health effects of SSBs) and creating cohesion in the nutrition community. Opportunities for the nutrition community to generate commitment for a SSB tax were increasing connections to key policy-makers including the ministry of finance (MoF), and by collaborating with other currently independent pro-SSB tax organisations, to pool resources and strengthen coalitions and advocacy in Australia.
- Countering the undermining influence of the food, beverage, and sugar industries may contribute to generating political commitment for a SSB tax. In particular we suggest strategies to reduce industry influence in policy decision-making, including strengthening restrictions on corporate lobbying and political financing. Addressing the concerns of the sugar industry, such as by hypothecating some SSB tax revenue to address economic difficulties of farmers, may also reduce their influence on political support and contribute to generating commitment for a SSB tax.

** Implications for the public**

Sugar-sweetened beverage (SSB) taxes are an effective nutrition policy that is underutilised globally. We studied factors both enabling and impeding the adoption of a SSB tax in Australia in order to understand ways of accelerating the adoption of this policy action. We show that the food and beverage industries are well connected and collaborate together in an effort to undermine support for a SSB tax, including via lobbying government, promoting self-regulation, and stressing the economic importance of their industries. Together with the presence of unconducive neoliberal ideologies and opposition from the Australian sugar industry, both major Australian political parties are unsupportive of a SSB tax. We provide a number of recommendations for ways to counter these undermining industry influences by increasing the transparency of government-industry relationships and thereby contribute to generating political commitment for a SSB tax, other nutrition issues, and issues more broadly that suffer from opaque government-industry relationships.

Unhealthy diets and poor nutrition are the most important preventable risk factors affecting health globally.^1^ Nevertheless, there has been limited global adoption of effective policies for their prevention.^2,3^ Leading international health authorities call on governments to adopt a comprehensive policy approach to improve nutrition, involving the adoption of multiple synergistic interventions, and a strong role for government and the use of law and regulation.^4-6^ One nutrition policy action that has recently gained worldwide momentum are sugar-sweetened beverage (SSB) taxes, which have been introduced in over 50 jurisdictions.^7^ SSBs are non-alcoholic beverages that contain caloric sweeteners such as carbonated soft drinks, energy drinks, sports drinks, milk-based drinks, juice drinks, and sweetened waters.^7^ Consumption of SSBs show strong dose-response associations with several non-communicable diseases including obesity,^8^ type 2 diabetes,^9^ and dental caries and erosion.^10^ One important political objective of SSB taxes is to reduce this disease burden by increasing retail prices, reducing consumption, generating public awareness, and incentivising non-price industry responses (eg, product reformulation).^7,11^ Whilst evaluations reveal SSB taxes to be effective in reducing purchases and intake of SSBs, SSB taxes are underutilized globally.^3,12^

In recent decades, a growing literature has examined the political economy of nutrition, including the power of different actors to drive or constrain nutrition policy reform, across diverse socio-political and economic contexts.^13,14^ This includes studies examining SSB tax policy processes in countries where SSB taxes were adopted, revealing several influential factors. These include: the organisation and cohesion of pro-SSB tax non-governmental organisations (NGOs) and civil society groups; employing resonating frames, such as revenue generation; beverage industry opposition; design and package of the tax; the level of historical discussion of SSB taxes; and emergence of policy windows including periods of fiscal policy reform.^15-23^ Recent literature also focuses on understanding the corporate political activity of ultra-processed food and beverage industries as an impediment to nutrition policy reform.^24-26^

The SSB tax policy process literature and the public health nutrition policy literature more broadly, primarily utilises retrospective policy analyses with a limited number of studies utilising prospective analyses to develop and inform advocacy strategies.^27^ Understanding the pre-SSB tax implementation barriers therefore, may help accelerate the global adoption of this underutilized nutrition policy.^13,28^

In Australia, per capita SSB consumption is among the highest in the world.^29^ Moreover poor nutrition in Australia incurs significant health system costs, constrains workforce productivity, and contributes to social inequalities. The annual financial cost of obesity to the Australian community for example, was an estimated $8.6 billion in 2011-2012.^30^ Whilst there is widespread support for a SSB tax from over 35 Australian public health organisations,^31^ the two major political parties – the Liberal Party of Australia (conservative, centre-right) and the Australian Labor Party (social-democratic, centre-left) – routinely reject calls to consider a SSB tax.^32,33^ Australia lacks a comprehensive food and nutrition policy. Instead, a ‘patchwork’ of policy measures have been adopted, focusing mainly on a voluntary front of pack labelling scheme (Health Star Rating), the reformulation of packaged foods, and the food industry’s self-regulation of its marketing practices.^34,35^

One study guided by the policy process theory Multiple Streams Theory, documented the following barriers for SSB tax adoption in Australia: industry influence, ineffective advocacy efforts from the pro-SSB tax community, limited political support for paternalistic policies, and limited pressure from civil society.^36^ This analysis however, relied on public information and experiences of relevant stakeholders were not explored. Other studies investigating the factors influencing regulatory interventions targeting obesity prevention in Australia found the following barriers: limited cohesion among public health groups, limited support for intervention within government, the ‘productivist’ power of industry groups, the complexity of the food policy-making structures, and a lack of evidence for interventions.^28,37^

 The present study aimed to gain deeper insight into the factors influencing commitment for a SSB tax in Australia, by drawing on interviews with key stakeholders and document analysis, and guided by a theoretical framework designed for this purpose. Several key questions are addressed: What factors enabled and/or impeded political commitment for a SSB tax in Australia? What future actions may help to generate commitment for such a tax, and for nutrition policy action more broadly in Australia and internationally?

## Methods

A case study design was adopted^38^ guided by a theoretical framework.^39^ Data were collected from semi-structured interviews with key stakeholders and triangulated with data from media articles, journal articles, and government and organisational documents to minimise bias.

###  Theoretical Framework

In studying health policy processes, adoption of conceptually and empirically informed theories can aid in understanding the complexities and identifying relevant influential factors.^40^

This study adopted a theoretical framework (the ‘Framework’) developed to specifically understand political commitment for nutrition.^39^ Political commitment here is defined as, “the intent and sustained actions over time by societal actors to achieve the objective of reducing and eliminating the manifestations and causes of (malnutrition).”^39^ The Framework was developed by synthesizing and modifying prior health policy process theories based on a review of the empirical nutrition policy process literature.^39^

The Framework hypothesizes 18 factors, conceptualised as increasing or decreasing the probability of political commitment.^39^ A description of each of these factors can be found in Table 1. The following factors from the Framework were excluded as limited data supported their relevance for the present study: International actors; Strength of institutions; Effective vertical coordination; Legislative, regulatory and policy frameworks; Strategic capacities; Organisational capacities; Financial resources.

**Table 1:** Factors Driving Political Commitment for Nutrition

**Category**	**Factor and Description**
Actors	(1) NAN effectiveness: effectiveness of NANs, the individuals and organisations operating within a given jurisdiction who shared common principles, causal beliefs and/or interest in tackling malnutrition and who acted collectively to do so.
	(2) Strength of leadership: presence of committed and politically savvy individuals, within or outside of government, recognised as strong champions for nutrition.
	(3) Civil society mobilisation: extent to which civil society groups mobilised to address malnutrition, including NGOs and social movements collectively representing the interests of citizens.
	(4) Supportive international actors: degree to which actors with an international scope of operations and/or membership initiated, championed and/or supported nutrition policy and programming responses.
	(5) Private sector interference: degree to which mobilised private interest groups undermined effective nutrition policy responses, including food producers, retailers, marketers and their representative peak bodies.
Institutions	(6) Strength of institutions: extent to which coordinating agencies and institutional systems mandated to address malnutrition were empowered to effectively coordinate multisector/multilevel responses and advocate for sustained attention and resources.
	(7) Effective vertical coordination: degree to which nutrition policies were effectively coordinated, implemented and monitored across levels of governance, particularly regarding the incentives of subnational actors to adopt, progress and benefit from central government policies.
	(8) Legislative, regulatory and policy frameworks: degree to which national nutrition policies, operational plans and enabling legislation were well-designed and enacted, and/or the alignment of nutrition objectives with broader policy agendas and regulatory frameworks.
Political and societal contexts	(9) Supportive political administrations: degree to which members of the executive (eg, head of state, ministers), legislative (eg, parliamentarians) and administrative (eg, agency heads, senior officials) branches of government initiated and championed nutrition responses.
	(10) Societal conditions and focusing events: extent to which changing societal conditions (long-duration phenomena) or focusing events (short-term processes) focused attention onto nutrition or closely related issues and presented opportunities or impediments to commitment-building.
	(11) Ideology and institutional norms: extent to which entrenched belief systems and practices predominant within political systems, policy-making institutions and/or in society-at-large, negatively skewed perceptions about malnutrition problems and undermined effective policy responses.
Knowledge, evidence, and framing	(12) Credible indicators and data systems: availability of credible indicators and high-quality data systems for monitoring nutrition problems, informing policy design, tracking progress and empowering accountability systems.
	(13) Evidence: extent to which robust evidence on the causes, manifestations and consequences of malnutrition and the efficacy and cost-effectiveness of interventions was available, clearly communicated and accepted.
	(14) Internal frame alignment: degree to which NANs were aligned around a common interpretation and narrative of a given malnutrition problem including its definition, magnitude, causes and solutions for resolving it.
	(15) External frame resonance: degree to which NANs publicly portrayed (ie, framed) nutrition problems and solutions in ways that resonated with and motivated action by external audiences, and countered the frames deployed by opponents.
Capacities and resources	(16) Strategic capacities: degree to which NAN members possessed ‘soft-power’ skills including the capacity to generate consensus, resolve conflicts, respond to recurring opportunities and challenges, build strategic alliances, undertake strategic communications and related tasks.
	(17) Organisational capacities: degree to which NAN members possessed the technical knowledge and skills, administrative systems and human resources required to generate commitment, including through the effective management of nutrition policy and programming responses.
	(18) Financial resources: degree to which nutrition budgetary commitments and financing systems incentivised multisector/multilevel coordination, ensured successful policy implementation and created ownership and entitlements among political elites, policy-makers, citizens and other stakeholders.

Abbreviations: NAN, nutrition actor network; NGOs, non-governmental organisations. Note: Adapted from Baker et al.^39^

There are a plethora of policy process theories that have been applied to examine public health policy processes, the most commonly used being Multiple Streams Theory and the Advocacy Coalition Framework.^39,40^ A description of these theories is beyond the scope of this paper, but see Sabatier and Weible,^41^ John^42^ and Gillespie and van den Bold^43^ for detailed overviews. The Framework was adopted over the other policy process frameworks and theories because it is a synthesis of three other policy process frameworks, namely Multiple Streams Theory, Shiffman and Smith’s Health Priority Setting Framework, and Heaver’s work on political commitment for nutrition.^39^ Synthesis theories have been described as superior to non-synthesis theories due to their ability to capture the complexity and multifaceted nature of the policy process.^41,42^ Moreover, unlike all other policy process theories the Framework was modified based on the empirical nutrition policy process literature which makes it particularly well suited for identifying the relevant factors involved in nutrition policy processes specifically.

###  Data Collection

Sixteen interviews were conducted between April 2020 and May 2020 with informants spanning the academic, government, civil society and industry sectors (Table 2). Informants were recruited using purposive snowball sampling strategy.^44^ Purposive sampling began by examining submissions to the Select Committee into the Obesity Epidemic in Australia established in 2018, as many organisations indicated their involvement in the SSB tax issue in their submissions.^45^ Relevant informants were those that are or were actively involved in the SSB tax issue in Australia. Interviews were conducted over Skype and lasted between 43 and 102 minutes. An interview guide was used which consisted of questions that were formed on the basis of the factors from the Framework.^39^ One pilot interview was conducted to refine the interview guide and interviews were recorded and transcribed verbatim. Informants were de-identified and presented using general descriptors (eg, Academic) to preserve their anonymity. Informants were given the opportunity to review transcripts for accuracy.

**Table 2:** Characteristics of Informants

**Position/Sector**	**No.**
Academic	3
Civil society (public interest NGO)	7
Government	3
Sugar industry	2
Beverage industry	1
Total	16

Abbreviation: NGO, non-governmental organisation.

 News articles were obtained from Factiva with searches restricted to 2010-2020, Australia, and “similar” duplicates. Government documents were sourced from ParlInfo with searches limited to 2010-2020, and restricted to ‘Australian Parliament Website,’ ‘House of Representatives,’ ‘Senate,’ ‘Committees,’ ‘Bills and Legislation,’ ‘Constitution,’ ‘Publications’ and ‘Library’ collections. Scholarly articles were sourced from Griffith University, PubMed and Google Scholar databases. Industry and organisational documents were sourced from their respective websites. Search terms included truncations and synonyms of ‘soft drink,’ ‘sugar-sweetened beverage,’ ‘sugary drinks,’ combined with ‘tax’ and ‘levy.’

###  Data Analysis

Data from all sources were subject to “codebook” thematic analysis.^46^ In this approach themes are conceptualised as “domain summaries,” ie, summaries of the data, and are generated deductively, ie, prior to analysis, using a codebook to guide coding.^46^ Briefly, this occurred over four stages.

 First, a codebook was developed to guide coding using the Framework’s political commitment factors as overarching themes. Second, using the codebook the data was coded by the lead author. Open coding was also utilised which allowed for the identification of codes that were not pre-determined by the codebook and theoretical framework. Third, sub-themes were generated by clustering codes with similar meanings in a hierarchical manner. Last, sub-themes were revised to accurately capture the meaning of the codes they represent. Data analysis was supported by the qualitative analysis software NVivo, Release 1.2.

## Results

###  Evidence of Political Commitment for a SSB Tax

From at least 2016 onwards, significant publications and activity by pro-SSB tax individuals and organisations were immediately followed by political expressions of no support for a SSB tax.^32,47^ Consequently, informants described political commitment for a SSB tax as low in Australia. For example;

“*There has never been any support from government, from Labor or the Coalition for a sugary drinks tax. The only support comes from the Greens”* (Executive manager, public health NGO).

“*Every time it’s been put on the table, we’ve seen a pretty clear, ‘no, we’re not considering that’ and including sometimes even from the opposition ….a tax is never an election winning strategy”* (Academic).

“*It’s quite clear there’s no political will across either side of government. The only place where there’s will is with the Greens. So there’s not a readiness now” *(Former public servant, State Government).

“*I’ve talked to politicians about this…they find it hard to see how they’d implement a tax. In Australia, taxes aren’t popular, so most of them generally would say they can’t see a pathway” *(Industry representative).

 We found evidence concerning 11 of the 18 factors listed in the framework, that explain what has generated and constrained commitment for a SSB tax in Australia, namely: Nutrition actor network (NAN) effectiveness; Strength of leadership; Civil society mobilisation; Private sector interference; Supportive political administrations; Societal conditions and focussing events; Ideology and institutional norms; Credible indicators and data systems; Evidence; Internal frame alignment; and, External frame resonance. We consider these factors in the following sections, organized under the main categories of the framework.

###  Actors

####  Nutrition Actor Network Effectiveness

In the first factor in the framework, NANs are described as “…*the individuals and organisations operating within a given jurisdiction who share common principles, causal beliefs and/or interest in tackling malnutrition, and who act collectively to do so.*”^39^ Similar terms like ‘policy community’ and ‘advocacy coalition’ are also used.^40^

 The NAN (hereon the ‘Network’) involved in the SSB tax issue in Australia consisted of individuals and organizations from academic and civil society sectors. Civil society groups included public health NGOs (eg, Public Health Association of Australia), consumer groups (eg, CHOICE), public policy think tanks (eg, Grattan Institute), and medical and other professional associations (eg, Australian Medical Association).

From early 2010s onwards, the Network engaged in several activities to enhance their collective effectiveness including: Generating relevant evidence supporting the economic,^48^ health,^49,50^ and public rationale^51^ for a SSB tax; awareness raising including mass media campaigns highlighting the negative health consequences of SSBs^52,53^; and advocacy including direct and indirect engagement with politicians and policy-makers (as reported by informants). Overall these efforts led some Network members to view their efforts in generating commitment for a SSB tax as “quite successful” (public health NGO manager) with everyone working “as well as they can” (policy officer, public health NGO).

Nevertheless, several factors worked against the collective efforts of the Network. The wider public health nutrition network was described as densely clustered together with limited links to politicians or bureaucrats compared to industry.^54^ Moreover, informants perceived engagement by Network members with finance ministers and treasurers to be lacking:

“*One thing still missing in Australia is talking more to the finance minister, the Treasury, about this as an opportunity to raise money. Especially in a post COVID-19 [coronavirus disease 2019] era” *(Academic).

 Resource limitations were also noted by Network members as influencing their collective capacity to generate commitment for a SSB tax:

“*The public health industry has very finite resources. That’s one problem we always encounter…Commercial industries can dedicate time, money, resources, people, to working on this all the time, in ways that the public health sector just can’t” *(Policy officer, public health NGO).

####  Strength of Leadership

Strong leaders can increase Network effectiveness through building relationships, generating consensus positions, and effective communication.^39^

The Obesity Policy Coalition and its Executive Manager Jane Martin, were identified by Network members as providing strong leadership and championing a SSB tax. The Obesity Policy Coalition with colleagues from Deakin University, generated Network cohesion and consensus statements,^31^ directly engaged government officials,^45,55^ funded research,^48^ and generated significant attention to a SSB tax and related obesity prevention issues in the media. The Public Health Association of Australia, and the Australian Medical Association were also identified by informants as significant SSB tax advocates.

####  Civil Society Mobilisation

Civil society refers to NGOs and social movements which collectively represent citizen’s interests. Mobilisation of these groups can enable commitment by raising public awareness and influencing government through advocacy.^39^

Several pro-SSB tax community organisations were involved in government advocacy including: Sugar By Half, That Sugar Movement, Sugar Free Smiles, Parents’ Voice, YMCA, Consumers Health Forum, CHOICE, Queensland Country Women’s Association, and Australian Council of Social Service (ACOSS).^45^ All but the latter two were involved in collaborative efforts with the Network around the SSB tax.^31^

Some informants however, commented on the limited collective effort these organisations made to raise awareness of a SSB tax through their membership or media. Attempts to ignite public mobilisation therefore, were primarily pursued and funded by disease-oriented NGOs via mass media campaigns.^52,53^ When the public are asked however, the majority are supportive of a SSB tax when proceeds are used for public health initiatives.^51^

####  Private Sector Interference

Private sector interest groups often strongly influence commitment through lobbying policy-makers, pre-emptively adopting self-regulation, framing policy debates, and disputing evidence.^39^

 Network members viewed food, beverage, and sugar industries as having powerful influences on the SSB tax issue with some viewing industry’s voice as “the most powerful.” The following details several activities food, beverage, and sugar industries engaged in to undermine support for a SSB tax.

Several industry groups established an industry coalition known as the “Sugar Roundtable of Associations” created to “proactively defend against any proposed [SSB] tax.”^56^ The coalition included, Australian Beverages Council, Australian Food and Grocery Council, Australian Industry Group, Australian Association of National Advertisers, Australian Sugar Research Alliance, and Canegrowers Association.^56^ One public health NGO manager called the coalition “a very, very significant development” as it was the first industry collaboration they had seen in response to obesity prevention policy in Australia.

Food, beverage, and sugar industries lobbied policy-makers directly and through involvement in inquiries and juries on obesity.^45,56-58^ For example, the annual report of the Australian Beverages Council^56^ stated it devoted “…significant resources to keeping a tax off the policy table of either the Government or Opposition, through direct engagement with key politicians” and attributes industry support to the presence of politicians at their annual board meetings at parliament house.

Food and beverage industries influenced the SSB tax evidence base by funding peer-reviewed literature showing decreasing consumption of SSBs among Australians.^59-61^ Moreover, they criticise and misrepresent SSB research.^45,62^

Food and beverage industries employed several strategic frames to undermine support for a SSB tax including: the scientific evidence for a SSB tax is contested; focussing on SSBs is unfair because sugar is in more than SSBs; individuals and parents are responsible for SSB consumption; and a SSB tax would create significant economic burden on sugar and beverage industries.^45,62,63^

 On the last point, contrary to beverage industry, political, and Network perspectives, the sugar industry’s primary concern with a SSB tax was not the direct economic impact but rather that it could formalize the demonisation of sugar, which could in turn negatively impact sugar based communities’ perceptions of the industry;

“*…the evidence suggests the impact on consumption would be potentially temporary, and for individual farmers the economic impact is not a primary concern, because we’re exporting 85% of it…[however] the industry would contract for other reasons, part of which is because they feel like they don’t have a community supporting them growing their product”* (Sugar industry representative).

The Australian Beverages Council, the Australian Food and Grocery Council, and Australian Association of National Advertisers, all promoted and adopted self-regulation in an attempt to pre-empt and undermine government regulation.^24,28^ Recently in June 2018, the Australian Beverages Council launched the Sugar Reduction Pledge which commits the majority of the non-alcoholic beverage industry to reduce sugar by 20 per cent on average across the industry portfolio from 2015-2025.^64^ The pledge was launched at a press conference in Canberra alongside federal Health Minister Greg Hunt who publicly endorsed it.^64^

This combination was described by Network members as a detrimental “watershed moment” which “really took the heat out of the discussion at a political level.” One academic called the pledge “a deliberate ploy by industry to try and stave off a tax.” The pledge is presented by the Australian Beverages Council as reducing the sugar content of beverages, despite flaws in its design and monitoring, and hired the public relations agency Sefiani to promote its self-regulatory efforts.^65^

Food, beverage, and sugar industries also extended their influence through political party donations. Between 2016-2020 for example, Coca-Cola Amatil, Australian Sugar Milling Council, and Australian Food and Grocery Council donated $241 911, $133 760, and $43 300, respectively to Liberal and Labor parties and their associated entities.^66^

Of the Australian Food and Grocery Council donations, most ($41 800) went to The Menzies Research Centre in 2017-2018, a think tank and associated entity of the Liberal Party. In 2017 the Menzies Research Centre published *Fat chance: Why sugar taxes won’t work*, which critiques several papers in favour of a SSB tax, and was used by the beverage industry to undermine the SSB tax evidence base.^45^

###  Political and Societal Contexts

####  The Role of Political Administrations, Ideology and Institutional Norms

Ideology and institutional norms, are entrenched belief systems and practices dominant within political systems, policy-making institutions and/or in society-at-large.^39^ Political administrations are members of executive and legislative branches of government, and high-level government administrators (eg, senior public servants), who can champion or block policy change.^39^

 Among the most important reasons mentioned by informants for why SSB tax commitment was low in Australia was the presence of neoliberal ideologies, for example;

“*At a high level, I think it’s still, this perception that diets are a matter of individual responsibility. That just comes up with everything we do” *(Academic).

“*One of the biggest push backs has always been, ‘it’s up to the individual and as long as they’re educated they’ll make an informed choice’”* (Public servant, State Government).

Both major political parties are unsupportive of introducing a SSB tax. The strongest opposition came from the Liberal-National Coalition, and centred around the Liberal party’s core ideology of individual freedom and free enterprise. Specifically, members of the Coalition argued Australia should not introduce a SSB tax because governments should play a minimal role in dictating Australians diets, that individuals and parents are responsible for what they eat and therefore interventions should focus on education, and governments should support industry self-regulation.^32,67,68^ For example,

“*Rather than attempting to manipulate Australian citizens through new taxes…governments…should be encouraging people to take greater personal responsibility for their lives” *(Senator James Paterson, Liberal Party of Australia).^68^

 Network members were aware of the Liberal-National Coalition’s strong opposition to a SSB tax and as such experienced a “slowdown in advocacy” and loss of “momentum” with their re-election in July 2019:

“*Right now, because of the lack of political appetite, we’re not doing a lot of work on it [SSB tax]. We’re not actively advocating for it publicly because that doesn’t seem to have been successful”* (Academic).

Conversely, Network members viewed Labor as more sympathetic to a SSB tax as it voiced a lack of a plan for a tax rather than outright rejecting one.^33,69^ Aligning with Labor’s core ideology of equal opportunity, concerns raised focused on the potential regressive nature of a SSB tax:

“*… certainly we are consuming too much sugar…we also need to look at the impact of taxes, who it is hitting and whether it is hitting working people who are already struggling to make ends meet. So we would look very cautiously at any proposal*” (Hon Anthony Albanese, Member of Parliament, leader of the Opposition).^69^

Whilst Labor’s Deputy Premier of WA Roger Cook supports a SSB tax,^45^ The Greens were the only political party supporting a SSB tax.^45^

Additional political concerns were expressed, especially from Coalition members, about the impact a SSB tax may have on the Australian sugar industry^67,70^:

“*Let’s not hurt our cane growers, who are already hurting due to low prices, with a government intervention which won’t help solve the problem”* (Hon David Littleproud, Member of Parliament, Deputy Leader of the National Party).^67^

Queensland and New South Wales, where the majority of sugarcane farmers and millers are located in Australia, have several political seats strongly contested by Labor and Liberal parties. The 2016 and 2019 elections had 8 and 2 marginal seats in federal electoral divisions respectively, containing sugarcane farms, mills, or other economic activity associated with the sugar industry.^71,72^

 Given the sugar industry’s opposition to a SSB tax, Network members believed political parties were unwilling to support a SSB tax to increase their chances of winning these marginal seats. Indeed, Network members described these marginal seats as having a “huge” and “surprising level of influence.”

####  Societal Conditions and Focussing Events

This factor refers to long-term changes in societal conditions and shorter-term focussing events bringing attention to the SSB tax issue.^39^

 Network members viewed several factors as bringing attention to the SSB tax issue in Australia including: the changing scientific narrative around the harms of excessive sugar consumption; rising rates of obesity in Australia and globally; lack of effectiveness in existing public health policies for obesity; and the spotlight from countries implementing SSB taxes, especially Mexico in 2014 and the United Kingdom in 2018, for example:

“*Even though some countries or jurisdictions had some kind of tax in place before 2014, the implementation of the tax in Mexico and then the subsequent evaluation, that’s when it really hit the spotlight [in Australia]”* (Academic).

 Recognizing the influence of a country’s economic position in its likelihood of SSB tax adoption, some Network members see an advocacy opportunity emerging given COVID-19’s economic impact in Australia.

###  Knowledge, Evidence, and Framing

####  Credible Indicators and Data Systems

This factor refers to monitoring nutrition problems and the associated data which can be used to generate attention, support advocacy, and inform policy implementation.^39^

Australia’s monitoring and surveillance system for food and nutrition was referred to by Network members as using “low quality” and “outdated” data^73^ and as “ad hoc and uncoordinated.”^74^ This is partly due to sparse and varied data collection methods of national nutrition surveys: there was a 17 year gap between the 1995 National Nutrition Survey and the 2011-2012 Australian Health Survey, and current surveys use less reliable self-reported data.^74^

These inadequacies extend to SSB consumption data which was only collected from three national health surveys in 1995, 2011-2012, and 2017-2018.^75^ These non-routine surveys and differences in methodology led to problems in reporting trends over time, including patterns in overweight and obesity.^76^

####  Evidence

This factor refers to the presence and communication of evidence on the determinants of nutrition problems and the effectiveness of proposed interventions.^39^

Several reviews and meta-analyses link SSB consumption to disease and a SSB tax to reduced consumption of SSBs.^8,9,12^ Moreover, Australian modelling studies demonstrated the economic and health savings of a SSB tax.^48-50^ Consequently, Network members interviewed believed the evidence base supporting a SSB tax in Australia was strong.

A diverging yet minority view however, was expressed by two academics publishing articles questioning the focus on SSBs by challenging the role added sugars play in the prevalence of obesity in Australia.^77,78^ This approach was heavily criticised by various Network members.^79^

Meanwhile, food and beverage industry’s claimed the evidence base supporting a SSB tax was insufficient with particular focus on inadequate real-world evidence linking SSB taxes to declining obesity rates.^45,47,63^ Drawing on industry’s arguments, this claim was echoed by some Coalition members.^45^ The absence of this evidence was explained by Network members as a result of relatively recent global adoption of SSB taxes giving insufficient time to evaluate their long term effects on obesity.^45^

 Accordingly, some Network members labelled industry’s claims of insufficient evidence as a “tactic” to “require unrealistic standards of evidence,” whilst others are sceptical of the difference this evidence will have on generating political commitment for a SSB tax:

“*All evidence is great and helps. Would that tip it over the line in Australia? I don’t think so. The evidence base is actually solid enough, and that’s not necessarily what this is about”* (Policy officer, public health NGO).

####  Internal Frame Alignment

Internal frame alignment refers to the degree to which the Network is aligned in their understanding and portrayal of the SSB tax issue, which can in turn influence commitment by influencing Network effectiveness.^39^

Recent Network collaboration led to the formation of coalitions which subsequently generated coherence within the Network by positioning the SSB tax as a priority policy solution.^31,80,81^*Tipping the Scales: Australian Obesity Prevention Consensus* for example, is a consensus document signed by 35 leading Network organisations in 2017, outlining 8 priority policy actions for obesity, including a SSB tax.^31^ Network members commented on the increased Network cohesion due to these coalitions:

“*Before the Tipping the Scales report [advocacy] was a bit disjointed, different public health groups were picking different things to advocate for. Some groups were focused on reformulation, some…on education campaigns, others pushing for regulations…The Tipping the Scales report and similar initiatives around that time, helped people to prioritize what the asks were*” (Academic).

 Consequently, there was widespread support for a SSB tax across the Network, for example;

“*It’s now pretty much accepted in the public health community that that’s [a SSB tax] probably our biggest bang for our buck from an obesity prevention point of view”* (Academic).

Whilst these coalitions represent the activity of 106 organisations and institutions, 20 other organisations were found to support a SSB tax which were not involved including, Arthritis Australia, ACOSS, Grattan Institute, Queensland Country Women’s Association, Australian College of Nursing, and Australian Medical Association.^45^

####  External Frame Resonance

This factor refers to how the Network publicly portrays or frames the SSB tax issue, and the extent to which this framing resonates with external actors, mobilizes supporters, and counteracts opponents.^39^

The Network primarily framed the SSB tax as a public health policy, specifically that a SSB tax could reduce intake of free sugars from SSBs which could in turn reduce the associated disease burden from non-communicable diseases.^82-84^ This focus on sugar and its impact on disease resonated with the Australian public according to an analysis of the LiveLighter mass media campaign for SSBs which utilised this framing and saw reductions in SSB consumption.^52^

 Moreover, since the United Kingdom announced its Soft Drinks Levy in 2016, Network members attempted to move away from the language of “tax” favouring “levy.” This decision was made by Network members to avoid the negative public and political association with taxes in Australia. Despite Network efforts however, the media predominantly referred to the policy as a “sugar tax.”

## Discussion

This study applied a framework for understanding the determinants of political commitment for nutrition, to reveal the factors enabling and impeding commitment for a SSB tax in Australia. It supplements findings from Sainsbury et al^36^ who used Multiple Streams Framework to study the politics of SSB taxation in Australia. Both studies agree on the influence of industry, limited political support, and unconducive ideologies. However, the present study reveals several further insights into Network activity and the politics of taxing SSBs in Australia, including how commitment for such a tax might emerge in the future. A summary of the findings can be found in Table 3. The following sections examine the factors that enabled and/or impeded political commitment for a SSB tax in Australia and in light of these findings how commitment for a SSB tax could be generated in the future.

**Table 3:** Summary of Enabling, Impeding, and Future Generative Factors Influencing Political Commitment

**Category**	**Enabling Commitment**	**Impeding Commitment**	**Generating Political Commitment**
Actors	Network with strong leadership, generating supportive evidence, raising awareness, advocating for action, collaboration with community organisations	Network is resource poor with limited links to government (especially finance ministers), and limited awareness raising from community organisations	Increase connections to key policy makers including MoF and treasury
		Strong, well connected, and collaborative influence of food and beverage industries who influence evidence base, employ strategic frames, promote self-regulation, and lobby government	Increase transparency of government and industry activity (eg, publish ministerial diaries, reduce federal donation threshold)
Political and societal contexts	The minor Greens Party supportive	Both major political parties unsupportive of SSB tax	Potential advocacy opportunity in change of government given opposition government is less opposed to SSB tax
	Supporting societal conditions including, rising rates of obesity, ineffective existing public health policies, international adoption of SSB taxes	Vocal sugar industry with marginal seats in sugar industry electoral divisions	Hypothecate some SSB tax revenue to address sugar industry concerns
		Presence of neoliberal ideologies	Frame SSB tax to align with dominant neoliberal ideologies
Knowledge, evidence, and framing	Strong intervention logic from evidence base	Poor monitoring and surveillance system for nutrition including SSB consumption data	Utilise alternative frames to garner support from policy makers (eg, emphasize revenue generation)
	Increased Network coherence via coalitions and consensus on SSB tax as a priority policy action	Lack of real-word evidence linking SSB taxes to declining obesity rates, and critical minority academic view questioning the focus on SSBs	Effectively communicate evidence base supporting SSB tax
	Public health policy framing of SSB tax resonating with public	Organisations supportive of SSB tax appear to be working separately from established coalitions	Collaborate with other pro-SSB tax organisations to increase opportunities for pooling resources and creating more effective alliances

Abbreviations: SSB, Sugar-sweetened beverage; MoF, ministry of finance.

###  Creating a More Effective SSB Tax Network

This section examines the factors associated with the Network including: NAN effectiveness; Strength of leadership; Civil society mobilisation; Internal frame alignment; and External Frame resonance. The Network engaged in several activities to generate political commitment for a SSB tax including, developing strong leadership, generating evidence, raising awareness, advocating for action, employing public health frames, collaborating with civil society organisations, and forming coalitions increasing their cohesion. Consequently, there is Network consensus that a SSB tax is a priority policy action. In contrast, previous studies documented limited leadership, a weak evidence base, and limited cohesion among obesity experts and advocates in Australia, indicating some progress was made towards a more effective Network.^28,36^

Several factors associated with the Network however, are likely impeding commitment for a SSB tax. First, compared to food and beverage industries the Network has less direct access to key policy-makers and therefore their comparative capacity to directly influence policy-makers and in turn policy including a SSB tax is lower.^54^ Not only is access to policy-makers important but which policy-maker is accessed is important. The agency responsible for SSB tax implementation is typically the ministry of finance (MoF) or taxation/revenue department and not surprisingly, engagement with these agencies have been influential in passing SSB taxes.^16,18,20^ In Samoa^18^ the SSB tax originated from within the MoF, in Chile^20,21^ it was driven primarily by the MoF, and in Mexico^16,20^ the MoF played an important role with SSB tax proponents engaging solely with the MoF. In Australia, the weak connections from the SSB tax Network with the MoF and Treasury therefore are likely impediments. Increasing connections to key policy-makers including MoF and Treasury therefore, may contribute to generating commitment for a SSB tax.

Second, powerful pro-SSB tax coalitions have been reported as influential for SSB tax adoption in Mexico, Chile, and Colombia.^16,20^ In Mexico for example, the pro SSB tax network formed an alliance of over 650 groups including NGOs, academics, and civil society groups whose effective collaboration included planned division of labor to cater for different organisational strengths.^16^ In Australia therefore, the identification of several organisations not included in Network coalitions indicates potential siloing of SSB tax advocacy and lost opportunities in pooling resources and creating stronger more effective alliances.^13,39^ Collaboration with some of these organisations (eg, ACOSS) may be fruitful given their proximity to some SSB tax concerns (eg, regressivity). Extending current coalitions to known SSB tax supporters therefore, may contribute to generating commitment for a SSB tax.

Third, whilst framing the issue around sugar and its impact on disease resonated with the public, limited resonance from policy-makers suggests the Network could benefit from placing more emphasis on alternative frames. One frame utilised in several countries where SSB taxes passed, including Mexico,^16,20^ France,^17^ Colorado,^19^ Fiji,^18^ Samoa,^18^ Philadelphia,^22^ and Finland,^15^ is emphasis on the tax to generate revenue. In Philadelphia for example, the SSB tax was deliberately not framed as a health intervention but rather a revenue generating policy that could finance universal prekindergarten.^22,23^ This focus on financing universal prekindergarten shifted the discussion away from popular anti-SSB tax arguments such as infringing on individual liberty.^22^ The attractiveness of the revenue generating frame was also enhanced in several countries due to the concurrent presence of economic shortfalls or crises.^15-19^ As Australia experienced its biggest economic contraction since the Great Depression due to COVID-19, emphasizing the additional annual $500 million a SSB tax can generate, including gains in productivity and economic savings in improved health may be influential in generating commitment.^39,85^ Moreover, this framing may be more appealing to concerns expressed by the sugar industry.

###  Political Leadership and Ideology 

 Beyond Network activity, limited political leadership and the presence of neoliberal ideologies were strong impediments to SSB tax commitment.

The strong opposition from the centre/right Liberal-National Coalition to the SSB tax is in contrast to other centre/right-wing parties in power where SSB taxes passed, such as France, the United Kingdom, Finland, and Denmark.^15^ Hagenaars et al^15^ suggests these conservative parties partly supported SSB taxes because the framing aligned with their conservative ideologies. The most persistent ideological barrier in this study were neoliberal ideologies, a common finding in the nutrition policy process literature.^13,39^

Specifically, industry and political opposition to the SSB tax most commonly drew on the idea that individuals should be responsible for their own diets, a view also common among SSB consumers.^86^ Therefore, to reduce opposition and potentially enhance commitment for a SSB tax, the Network could engage with these dominant ideologies by reframing the SSB tax to align with them.^15^ For example, by framing the SSB tax as promoting autonomy by helping consumers make informed choices, as new SSB prices will reflect their true price on health and healthcare. Otherwise, an advocacy opportunity may present itself in a change of government, given Labor is less opposed to a SSB tax, has some supportive members, and historically stronger commitments to preventative health and obesity.^28^

###  The Power of Industry Groups

The considerable power of food and beverage industries to undermine SSB tax commitment as found in this study has been documented in several other countries,^13,19,20,26^ and for other nutrition issues in Australia.^25,28^ In Fiji, organised beverage industry opposition led to the removal of the domestic SSB tax.^18^ Moreover, a less vocal beverage industry in Colorado^19^ and a disorganized response from the beverage industry in France^17^ have been attributed as important factors enabling SSB tax adoption in these countries. In Australia, many of the strategies used by the food and beverage industry to undermine SSB tax commitment as documented here, have been employed by these industries for other nutrition issues.^25,87^ Given the significant influence the food and beverage industries have on commitment for nutrition policies, approaches are needed to counteract or limit their influence. Several recommendations are given based on this study’s findings.

While evidence of industry lobbying was found, its full extent is disguised by inadequate transparency. Creating a more effective lobbyist register, and publishing ministerial diaries (as was done in Queensland, New South Wales, and Australian Capital Territory) could enable greater monitoring of relationships between industry and government, or lack thereof for the Network.^88^ Moreover, given 50% of money received by Liberal and Labor parties is not publicly accounted for, reducing the Federal donation threshold and requiring multiple donations from the same donor to be aggregated could allow greater tracking and exposure of industry influence on political relationships and power.^89^

Industry sponsorship of academic research, as documented in this study, can bias conclusions and drive research agendas away from questions relevant for public health.^90^ This could be limited by increasing transparency through heightening disclosure of funding sources and conflicts of interests and strict guidelines to regulate industry interaction with research institutes.^90^

The sugar industry’s strong political influence on SSB tax support is also cause for consideration. Whilst their influence may be waning given the reduction of marginal seats in sugar electoral divisions in the 2019 Federal election, addressing sugar industry concerns may increase political support. For example, some SSB tax revenue could be used to address any economic challenges in converting lost domestic sugar sales to international markets.^91^ Addressing the sugar industry’s social concerns however are more challenging, but may benefit from funds to assist in increased diversification (eg, ethanol production) and potentially crop substitution.^92^

###  Enabling Societal Conditions

In several countries, the presence of economic shortfalls or crises, or policy windows linked with wider financial policy reform, enabled SSB tax progression.^15-19^ Prior to COVID-19, this was unlikely influential for Australia given 1991-2018 saw the longest stretch of uninterrupted gross domestic product growth ever recorded in the developed world.^93^

 Thus, Australia may be slower to consider a SSB tax given it was in a more privileged economic position and hence lacking economic pressures to generate government revenue. Although, as suggested by some informants the economic impact of COVID-19 may create an advocacy window.

###  Utility of the Framework and Future Research

The factors identified by previous studies as important for SSB tax adoption, as drawn upon here, fit within the Framework’s conceptualisation of factors that contribute to political commitment.^15-22^ Moreover, the Framework adopted in this study identified factors that received little to no attention when Multiple Streams Theory was adopted by Sainsbury et al,^36^ most notably, findings from the ‘Credible indicators and data systems’ and ‘External frame resonance’ factors. This speaks to the utility of the Framework for analysing nutrition policy processes.

Given most countries have not introduced SSB taxes, future prospective policy analyses of SSB taxes could support nutrition networks in other countries to more effectively engage with policy processes. Whilst the Framework has proven useful for analysing nutrition policy processes, future studies could help to elucidate the complexities of each of these 18 factors, to facilitate how best to understand and engage with them. Some progress has been made here with the systems dynamic approach to understanding NAN effectiveness^94^ and the framework developed by Mialon et al^95^ for monitoring the corporate political activity of the food industry.

###  Limitations

There are several limitations of this study. As with all interpretive research involving a single case study design and qualitative data, the results should be interpreted with caution, and should not be generalised to other jurisdictions.^38^ Moreover, the relative importance of the factors in driving political commitment is undetermined. This was addressed by asking Network members to give their perspective on the most important factors impeding commitment for a SSB tax.

 The results indicate Network members believe food, beverage, and sugar industry activity, unsupportive political parties, and neoliberal ideologies, are the biggest impediments explaining low political commitment for a SSB tax in Australia. Consequently, focussing on these factors may be more fruitful for understanding and generating commitment for a SSB tax and similar nutrition issues. However, this approach to weighting findings should be interpreted with caution given the difficulty in assessing the relative importance of factors beyond Network perceptions.

 Civil society (public interest NGO’s) comprised the largest share of informants which may represent a bias in favour of civil society perspectives. Moreover, the data was coded by the lead author only, which may influence the reliability of the findings. Finally, as the study was specifically focussed on SSB tax policy processes it did not consider alternative solutions to the SSB issue or other malnutrition issues that other stakeholders may have been involved in.

## Conclusion

 The activity of the SSB tax Network led to significant progress towards generating political commitment for a SSB tax. The identification of several impediments, many of which are common to other nutrition issues in Australia and around the world, provides an explanation for why political commitment for a SSB tax is low in Australia and reveals several opportunities for how it might be generated in the future. Particularly, the strong and persistent influence of the private sector on the SSB tax issue and many other nutrition issues, indicates further research is needed to understand the effectiveness of different approaches to counter these undermining influences.

 This study supports the utility of the Framework for analysing the determinants of political commitment for nutrition. The Framework was comprehensive in its scope, with no additional factors identified, and detailed in its description of each factor and their interconnected relationships.

## Acknowledgements

 The authors would like to thank Jessica Lee and Ellie Martus for preliminary guidance of this research.

## Ethical issues

 Ethical approval was granted by Griffith University Human Research Ethics Committee (GU 2020/119). Recruitment was voluntary and all participants were provided with an information sheet regarding the project and signed a consent form, including consent to publish.

## Competing interests

 Authors declare that they have no competing interests.

## Authors’ contributions

 TD wrote and revised the paper, led the conceptual design of the study, the collection, analysis, and interpretation of data, and carried out all of the ethics review procedures. PB contributed to the conceptual design of the study, reviewed, edited, and provided comments on drafts, and provided supervision.

## Disclaimer

 The views expressed in this article are those of the authors.

## Funding

 This research did not receive any specific grant from funding agencies in the public, commercial, or not-for-profit sectors.
